# Mineral nutrient variability of potato (*Solanum tuberosum* L.) tubers with different colors grown in Niksar, Kazova and Artova locations of Tokat Province, Turkey

**DOI:** 10.7717/peerj.15262

**Published:** 2023-06-09

**Authors:** Yasin Bedrettin Karan

**Affiliations:** Department of Field Crops, Tokat Gaziosmanpasa University, Tokat, Merkez, Türkiye

**Keywords:** Potato, Flesh color, Macronutrient, Micronutrient, Potassium

## Abstract

Potato is one of the most commonly consumed non-grain staple food crops in the world therefore, the mineral nutrient content of the potato is extremely important for human nutrition. The lack of mineral nutrients causes significant health problems, thus, many of these nutrients are often taken as supplements. This study was carried out to investigate the effects of potato flesh color and location on different mineral nutrient contents under Niksar, Kazova and Artova locations in Tokat Province, Turkey, during 2013 and 2014 potato growing seasons. The experimental design in each location was randomized blocks with three replications. In this study, a total of 67 clones (including varieties and advanced breeding selections) with nine white, 10 cream, 30 light yellow, and 18 dark yellow flesh colors were used. Potatoes with cream flesh colors had the highest K (23.81 g kg^−1^), P (0.31 g kg^−1^), Mg (1.20 g kg^−1^), Zn (27.26 mg kg^−1^), Cu (8.28 mg kg^−1^) and Mn (7.21 mg kg^−1^) contents, and the lowest Ca (45.6 mg kg^−1^) content. The mineral contents (except K and Cu) of potatoes grown in Artova were higher compared to the other two locations. The results clearly suggested that Artova is the most suitable location to produce potatoes with a high mineral composition, and Kazova is suitable to cultivate potatoes with high K and Cu contents. In addition, the knowledge of nutrient rich potato accessions is valuable for developing biofortified potato genotypes.

## Introduction

The human body needs mineral nutrients to perform its regular functions; therefore, the lack of these essential nutrients causes mineral malnutrition (White & Brown, 2010). The potato originated in the Andes Mountain and was domesticated during the pre-Columbian period over 8,000 years ago (Quiroz et al., 2018), and it is now the third most important staple crop in the human diet after wheat and rice (Changan et al., 2020). The potato has been a valuable source of food for human beings for a long time and also contributes to alleviating hunger due to its considerably high yield per unit area compared to many other crops (Tolessa, 2018). The potato is well-known for its high potassium and low sodium content, which makes it a potential food crop to include in a human diet to manage hypertension (Beals, 2019).

The processing quality of potato is closely related to its mineral composition, which is affected by the characteristics of the production area, the cultivars used in the production, soil and climate of the region, agricultural practices such as fertilization, irrigation, *etc.*, and storage conditions (Arvanitoyannis, Vaitsi & Mavromatis, 2008). In addition, Lal et al. (2020) reported that the micronutrient contents of potato cultivars are significantly influenced by the conditions of the growing environment. Soil characteristics affect the uptake of nutrients; thus, the mineral content in potato tubers may differ with the changes in soil environment. Hajŝlová et al. (2005) reported higher micronutrient contents in potato cultivars grown organically compared to those grown conventionally. Therefore, this study was conducted in three different locations to determine the effects of location on the mineral composition of potato accessions with four different flesh colors.

The potato, rich in mineral nutrients (potassium (K), phosphorus (P), iron (Fe), zinc (Zn) and other micronutrients), helps to alleviate micronutrient deficiency. Unlike the cereals, the bioavailability of Fe in potatoes is high due to very low levels of phytic acid (Navarre, Goyer & Shakya, 2009). A significant positive correlation was reported between potato consumption and recommended Fe and Zn intakes in children (Creed-Kanashiro et al., 2016). In addition, the potato is rich in many other useful contents, like some vitamins (C, B6, B3) and phenolic compounds (Burlingame, Mouillé & Charrondiere, 2009).

Genetic diversity of potatoes compared to many other crops is higher; therefore, potatoes can be cultivated under a variety of environmental conditions. In addition, high genetic variability enables potato to serve a valuable resource to enhance nutritional contents of potato tubers through breeding (Navarre, Goyer & Shakya, 2009; Singh et al., 2022a). Furthermore, differences in the macro- and micronutrient contents of potato cultivars can be used to identify molecular markers associated with mineral concentrations (Singh et al., 2022b). Similarly, White et al. (2009) stated that the mineral nutritional contents of crops, especially food crops such as potatoes, can be improved by breeding to obtain better cultivars or agricultural practices like mineral fertilizer application. Aside from genetic influences on mineral content, the growing environment has a significant effect on potato mineral nutrient composition. The highest K and Mg concentrations in potato tubers were obtained in urea applications among six different nitrogen fertilizer sources in Sao Paolo State, Brazil (Souza et al., 2022). The flesh color of potatoes is affected by the growing environment, the climate of the region, fertilizers used during the growing period, as well as pest and disease control practices affecting the health conditions of plants. However, the most important determinant of flesh color is inheritance (Von Rathlef, 1934), which has not been adequately explained or investigated. One of the rare studies on the flesh color of potato cultivars, Haynes et al. (2011) determined the carotenoid concentrations in some diploid potatoes. The researchers indicated that the carotenoid concentrations of diploid potatoes were almost 22 times higher than those in white flesh-colored potatoes. In another study, Kotíková et al. (2016) showed that the mean total carotenoid content of yellow flesh-colored cultivars was significantly higher than that of red/purple flesh-colored potatoes. The mineral nutrient contents of sweet potato cultivars with different colors have been studied (Aywa, Nawiri & Nyambaka, 2013; Gurmu, Hussein & Laing, 2017; Ayimbire et al., 2020), while, studies determining the mineral contents of colored potato tubers under different geographical locations have not been found in the literature. In this study, the effect of location, which differed in altitude, basic soil properties, and climate, on the mineral nutrient contents of four different colored potato clones was investigated. Potato breeders who want to breed nutrient rich potato cultivars would benefit greatly from knowing which potato accessions have the highest mineral nutrient content.

## Materials & Methods

### Site description

The field experiments were conducted in three different locations in Tokat Province, Turkey. The experiments in Niksar were located at 40°32′41″N latitude and 36°55′31″E longitude in 2013 (280 m asl) and 40°35′02″N latitude and 36°52′01″E longitude (298 asl) in 2014. The experimental field in Kazova was situated between 40°19′54″N latitude and 36°28′23″E longitude (596 m asl) in the first year, and between 40°19′58″N latitude and 36°28′25″E longitude (578 m asl) in the second year. The experimental field in the Artova was situated between 40°08′00″N latitude and 36°19′33″E longitude (1185 m asl) in the first year, and between 40°08′15″N latitude and 36°19′45″E longitude (1190 m asl) in the second year. In this study, a total of 58 potato clones with eight white, nine cream, 25 light yellow, and 16 dark yellow flesh colors, and a total of nine commercially registered potato varieties with one white, one cream, five light yellow, and two dark yellow colors were used. The field experiments were carried out in 2013 and 2014 growing periods. Physical and chemical characterization of soil samples collected from experimental fields were performed in the laboratories of the Soil Science and Plant Nutrition Department at Tokat Gaziosmanpasa University. The results of the soil analysis are given in Table 1.

The mean monthly temperature and mean total precipitation in experimental locations for 2013 and 2014 growing seasons and long-term are given in Table 2. The temperatures in all locations increased and peaked in August, then decreased until the harvest in October. The lowest mean temperatures were recorded in March, and the Artova location had the lowest mean temperatures (5.2 °C and 5.3 °C in 2013 and 2014, respectively) during the experiments (Table 2). Overall, the temperature in Artova was lower in all months of the growing seasons compared to the temperatures in Niksar and Kazova locations. Total precipitations during vegetation periods were similar to the long term averages in 2014, while the precipitation in 2013 was lower in Kazova and Artova and almost 50% higher in Niksar compared to the long term averages.

**Table 1:** Some of physical and chemical characteristics of experimental soils.

**Properties**	**Niksar**		**Kazova**		**Artova**	
	**2013**	**2014**	**2013**	**2014**	**2013**	**2014**
pH	8.13	8.09	8.29	8.33	7.79	7.88
Total Salt (%)	0.85	0.60	0.33	0.25	0.24	0.26
Clay (%)	45.2	36.1	31.9	25.5	24.9	23.7
Sand (%)	21.9	17.1	32.7	38.2	52.3	71.0
Silt (%)	32.3	48.0	35.1	35.5	25.8	25.8
CaCO_3_ (%)	40.8	20.9	23.1	22.2	8.5	5.0
P_2_O_5_ (kg ha^−1^)	172.0	129.0	70.1	71.0	73.5	55.8
K_2_O (kg ha^−1^)	1165.2	1975	1294.9	1278.7	477.1	869.0
Organic Matter (%)	3.48	2.99	2.00	2.59	2.31	2.09

**Table 2:** Climate data of the experimental locations.

**Months**	**NİKSAR (Altitude 280 for 2013 and 298 m for 2014)**						**KAZOVA (Altitude 578 m)**					
	**Mean Temp. (°C)**			**Total precipitation (mm)**			**Mean Temp. (°C)**			**Total precipitation (mm)**		
	**2013**	**2014**	**Long Term**	**2013**	**2014**	**Long Term**	**2013**	**2014**	**Long Term**	**2013**	**2014**	**Long Term**
March	11.1	11.1	9.1	26.3	43.2	43.2	9.7	11	6.6	20.2	43.4	38.1
April	14.5	15.8	14	54.3	9.7	67.2	13.7	16.2	11.8	35.2	13.2	56.1
May	19.6	18.4	17.5	69.4	67.3	63.5	19	17.4	15.1	21.2	33.6	57
June	21.5	21.3	20.8	89.4	73.5	45.4	20.8	20.3	18.3	23.0	61.4	35.8
July	22.7	24.8	23.3	50.1	10.2	17.0	21.9	24.2	20.7	1.2	7.0	10
August	23.3	25.7	23.1	41.2	0.04	9.1	22.5	25.2	20.5	0.4	1.0	6.6
September	19.0	20.8	20	60.7	39.2	22.9	18	20.2	16.4	11.6	39.0	16.6
October	13.3	15.1	15.1	52.4	57.7	49.2	11.7	14.1	11.8	41.8	47.0	33.5
Mean/Total	18.13	19.13	17.86	443.80	300.84	317.50	17.16	18.58	15.15	154.60	245.60	253.70

**Months**	**ARTOVA (Altitude 1200 m)**					
	**Mean Temp. (°C)**			**Total Precipitation (mm)**		
	**2013**	**2014**	**Long Term**	**2013**	**2014**	**Long Term**
March	5.2	5.3	2.8	28.7	55.7	45.2
April	9.3	10.2	8.5	44	20.4	63.4
May	14.4	12.7	12.5	14.6	62.6	65
June	16.0	15.7	15.7	19.9	36.8	38.1
July	16.9	19.5	17.9	0.8	2.5	8.9
August	17.4	20.5	18.0	0.0	6.2	3.1
September	13.1	15.6	14.4	13.5	47.6	11.5
October	7.2	10.0	9.4	45.4	77.0	38.0
Mean/Total	12.44	13.69	12.40	166.90	308.80	273.20

**Notes.**

Source: T.R. Ministry of Environment, Urbanization and Climate Change, General Directorate of Meteorology.

The research material is composed of 58 outstanding clones obtained from the International Potato Research Center (CIP). The cultivars used in the study were Serrana × 104.12LB; MF-1 × TS-4; Serrana × TS-9; Granola × TS-2; Serrana × DTO-33; Serrana × LT-7; Serrana × TS-4; Serrana × TPS-113; Serrana × TPS-67; MF-1 × LT-7; Pentland Crown × TS-2; and Granola × Huincul and Achrina × LT-7 potato hybrids. These 58 clones were classified under four flesh color groups as white (eight), cream (nine), light yellow (25) and dark yellow (16). In addition, nine commercially registered varieties (Agata, Marabel, Agria, Marfona, Granola, Lady Claire, Başçiftlik White, Slaney and Hermes) were used as the standard varieties. These 58 clones were classified in four flesh color groups, which were white (eight clones), cream (nine clones), light yellow (25 clones) and dark yellow (16 clones) (Table 3).

**Table 3:** The potato clones and pedigrees used in the experiment.

**No**	**Clone**	**Pedigree**	**Flesh color**	**No**	**Clone**	**Pedigree**	**Flesh color**
1	*A1/58*	*Serrana*×*104.12LB*	*WHITE*	30	*A3/26*	*Serrana*×*TS-9*	*LIGHT YELLOW*
2	*T5/32*	*Serrana*×*DTO-33*			31	*A3/321*		*Serrana*×*TS-9*	
3	*T5/4*	*Serrana*×*DTO-33*			32	*A3/164*		*Serrana*×*TS-9*	
4	*A5/70*	*Serrana*×*DTO-33*			33	T4/4*		*Granola*×*TS-2*	
5	*A5/6*	*Serrana*×*DTO-33*			34	*T6/3*		*Serrana*×*LT-7*	
6	*A8/34*	*Serrana*×*TPS-113*			35	*A6/119*		*Serrana*×*LT-7*	
7	*A13/3*	*Pentland Crown*×*TS-2*			36	*T6/17*		*Serrana*×*LT-7*	
8	*A13/1*	*Pentland Crown*×*TS-2*		37	*A6/71*	*Serrana*×*LT-7*		
9	*A3/116*	*Serrana*×*TS-9*	*CREAM*	38	*A6/103*	*Serrana*×*LT-7*		
10	*A4/9*	*Granola*×*TS-2*			39	*T6/28*		*Serrana*×*LT-7*	
11	*A5/60*	*Serrana*×*DTO-33*			40	*A8/11*		*Serrana*×*TPS-113*	
12	*A5/98*	*Serrana*×*DTO-33*			41	*T9/11*		*Serrana*×*TPS-67*	
13	*T5/14*	*Serrana*×*DTO-33*			42	A12/5		Achrina X LT-7
14	*A6/76*	*Serrana*×*LT-7*			43	*A2/11*	*MF-1*×*TS-4*	*DARK YELLOW*
15	*A7/12*	*Serrana*×*TS-4*			44	*A2/73*	*MF-1*×*TS-4*		
16	*A10/15*	*MF-1*×*LT-7*			45	*A3/110*	*Serrana*×*TS-9*		
17	*T11/10*	*Granola*×*Huincul*		46	*A3/223*	*Serrana*×*TS-9*		
18	*T1/26*	*Serrana*×*104.12LB*	*LIGHT YELLOW*	47	*A3/368*	*Serrana*×*TS-9*		
19	*A1/9*	*Serrana*×*104.12LB*			48	*A3/351*	*Serrana*×*TS-9*		
20	*A2/127*	*MF-1*×*TS-4*			49	*A3/142*	*Serrana*×*TS-9*		
21	*A2/99*	*MF-1*×*TS-4*			50	*A3/15*	*Serrana*×*TS-9*		
22	*A3/177*	*Serrana*×*TS-9*			51	*A3/346*	*Serrana*×*TS-9*		
23	*A3/29*	*Serrana*×*TS-9*			52	*A3/108*	*Serrana*×*TS-9*		
24	*A3/337*	*Serrana*×*TS-9*			53	*A3/74*	*Serrana*×*TS-9*		
25	*A3/275*	*Serrana*×*TS-9*			54	*A3/206*	*Serrana*×*TS-9*		
26	*A3/234*	*Serrana*×*TS-9*			55	*A9/8*	*Serrana*×*TPS-67*		
27	*A3/37*	*Serrana*×*TS-9*			56	*T9/13*	*Serrana*×*TPS-67*		
28	*A3/167*	*Serrana*×*TS-9*			57	*A9/4*	*Serrana*×*TPS-67*		
29	*A3/189*	*Serrana*×*TS-9*		58	*T10/8*	*MF-1*×*LT-7*		

### Experiment design and production practices

The experimental design in each location was a randomized complete block with three replications. Clones with different flesh colors were tested separately from standard cultivars with the same flesh color. The experimental plots consisted of two rows with a length of six meters. Twenty tubers were planted in each row. The materials used as seeds were produced in Artova and stored in a dark environment in cold air warehouses set to 4  °C, 85–90% relative humidity in the Faculty of Agriculture, Department of Field Crops, Tokat Gaziosmanpasa University. The tubers in Niksar were planted March 5, 2013 and March 1, 2014, in Kazova on April 15, 2013 and April 17, 2014, and in Artova on May 1, 2013 and May 9, 2014. Planting densities in all locations were 70 × 30 cm. Fertilizer was applied at a rate of 120 kg N, P and K per ha. All of P and K were applied during sowing, and 80 kg ha^−1^ of N was applied during the beginning of tuber formation. Plants were irrigated as needed using a drip irrigation system to maintain an adequate moisture level in the root zone. All necessary maintenance needed for potato cultivation were carried out properly. Weeds and insects were controlled by cultural and chemical practices. Metribuzin (Sencor® Bayer Crop Science) was used for weed control after emergence. Each plot received four sprays of insecticide (Decis, active ingredient, 2.8% (w/w) deltamethrin; Bayer Crop Science, Germany) to control Colorado potato beetle (*Leptinotarsa decemlineata).*

### Analysis of potato tubers for mineral compositions

Each year, tubers of each clone were harvested and carefully washed with tap water to remove soil, *etc*. Tubers were peeled completely, and for the second time, rinsed with tap water before slicing. The sliced tubers were kept in distilled water, and 20 ml of HCl acid was added to each sample. Tubers were then washed with distilled water two more times and left to dry on paper towels. Dried samples were ground when they reached a constant weight in the oven.

Mineral nutrient contents of tubers harvested in 2013 and 2014 were analyzed following the second growing season of the experiment. All analysis were performed in the laboratories of Soil Science and Plant Nutrition Department of the Agricultural Faculty at Tokat Gaziosmanpasa University, Tokat, Turkey. Each sample was analyzed three times for all nutrients. 0.2 g of ground tuber sample was weighed and placed into incinerator bottles. The samples were burned in a muffle furnace at 550  °C for at least six hours and left to cool to room temperature. The acid was evaporated on a hot-plate by adding two ml of HCl to the cooled samples. Then, two ml of HCl and 18 ml of distilled water were added to the samples again, and the final volume was made up to 20 ml. The samples were shaken well and filtered using blue band filter papers. Concentrations of K, P, Ca, Mg, Fe, Zn, Cu and Mn were determined using a Perkin Elmer inductively coupled spectrometry (Benton-Jones, 1989).

### Statistical analysis

The statistical analyses of the data were carried out in three steps. In the first step, normality of the data was tested before the analysis of variance (ANOVA), which indicated a normal distribution for nutrient contents; therefore, the ANOVA was carried out without any data transformation. The factors tested in the experiment were location (three locations), flesh color of potatoes (four flesh colors), and year (two growing seasons). In the second step, a three-way ANOVA was used to test the difference between colors, locations, and growing seasons. In the third step, when the ANOVA indicated a significant difference between the treatments, a least significant difference (LSD) test at a 5% probability level was used as post hoc to separate the means of nutrient contents. The nutrient content was expressed as the mean ± standard error of the mean. The statistical analyses were performed using SPSS version 21.0 (IBM 2012).

## Results

The mineral nutrient contents of potato accessions in two different growing seasons are given in Table 4. The nutrient contents, except phosphorus (P) and zinc (Zn), were significantly different between the growing seasons. Potassium (K), P, Zn and magnesium (Mg) contents of potatoes were higher in the first growing season, while calcium (Ca), iron (Fe), copper (Cu) and manganese (Mn) contents of potato accessions were higher in the second growing season. The potassium concentration of potato accessions in the first and second growing seasons was 28.62 and 15.32 g kg^−1^, respectively (Table 4).

**Table 4:** Mean nutrient contents (mean ± standard error) of all potato accessions in two different growing seasons (*N* = 603).

	K	P	Ca	Mg	Fe	Zn	Cu	Mn
	g kg^−1^				mg kg^−1^			
2013	28.62 ± 0.35^a*^	3.17 ± 0.03^a^	0.32 ± 0.00^b^	1.20 ± 0.01^a^	18.56 ± 0.22^b^	26.16 ± 0.31^a^	7.61 ± 0.07^b^	6.53 ± 0.05^b^
2014	15.32 ± 0.19^b^	3.18 ± 0.03^a^	0.35 ± 0.00^a^	1.16 ± 0.01^b^	33.41 ± 0.4^a^	25.65 ± 0.36^a^	8.33 ± 0.08^a^	7.44 ± 0.06^a^
CV-2013 (%)	29.6	22.8	28.4	19.9	29.2	29.0	21.3	18.9
CV-2014 (%)	29.7	20.3	27.2	23.6	33.9	34.5	23.6	20.3
*P* Value	0.001	0.654	0.001	0.001	0.004	0.242	0.001	0.001
LSD (0.05)	0.614	0.072	0.01	0.024	0.818	0.764	0.196	0.149

**Notes.**

* Means followed by different letters are significantly different by each other *P* < 0.05.

CV Coefficient of variation (%)

The mineral nutrient contents of potato accessions in three different locations are given in Table 5. The levels of all minerals in three different locations significantly (*p* < 0.01) varied from each other. The highest P, Ca, Mg, Fe, Zn and Mn contents in potato accessions were recorded in location three (Artova), which was characterized by higher altitude, lower CaCO_3_, pH, and clay content compared to the other locations (Table 1).

**Table 5:** Mean nutrient contents (mean ± standard error) of all potato accessions in two growing seasons (*N* = 603), three locations and four flesh colors (*N* = 542).

*N* = 603		**2013**	**2014**	**CV-2013**	**CV-2014**	***P* Value**	**LSD**
K	g kg^−1^	28.62 ± 0.35^a*^	15.32 ± 0.19^b^	29.6	29.7	0.001	0.614
P			3.17 ± 0.03^a^	3.18 ± 0.03^a^	22.8	20.3	0.654	0.072
Ca			0.32 ± 0.00^b^	0.35 ± 0.00^a^	28.4	27.2	0.001	0.01
Mg		1.20 ± 0.01^a^	1.16 ± 0.01^b^	19.9	23.6	0.001	0.024
Fe	mg kg^−1^	18.56 ± 0.22^b^	33.41 ± 0.4^a^	29.2	33.9	0.004	0.818
Zn			26.16 ± 0.31^a^	25.65 ± 0.36^a^	29	34.5	0.242	0.764
Cu			7.61 ± 0.07^b^	8.33 ± 0.08^a^	21.3	23.6	0.001	0.196
Mn		6.53 ± 0.05^b^	7.44 ± 0.06^a^	18.9	20.3	0.001	0.149

*N* = 402		**Niksar**	**Kazova**	**Artova**	**CV-Nk**	**CV-Kz**	**CV-Ar**	***P* value**	**LSD**
K	g kg^−1^	21.03 ± 0.51^b*^	24.25 ± 0.059^a^	20.67 ± 0.24^b^	48.2	48.5	23	0.001	0.752
P			2.91 ± 0.03^c^	3.2 ± 0.04^b^	3.39 ± 0.03^a^	21.9	22	18.2	0.001	0.088
Ca			0.33 ± 0.01^b^	0.32 ± 0.01^b^	0.35 ± 0.01^a^	29.5	27.8	26.7	0.001	0.012
Mg		0.99 ± 0.01^c^	1.21 ± 0.01^b^	1.34 ± 0.01^a^	21	16.1	17.3	0.001	0.029
Fe	mg kg^−1^	20.85 ± 0.37^b^	28.33 ± 0.68^a^	28.79 ± 0.59^a^	34.5	48.4	40.8	0.001	1.001
Zn			24.99 ± 0.48^b^	24.39 ± 0.31^b^	28.34 ± 0.41^a^	38.5	25.2	28.7	0.001	0.936
Cu			7.73 ± 0.10^b^	8.29 ± 0.08^a^	7.88 ± 0.09^b^	25.5	20.1	23.3	0	0.24
Mn		6.48 ± 0.07^b^	7.17 ± 0.07^a^	7.31 ± 0.08^a^	21.2	19.3	20.4	0.001	0.182

*N* = 542		**Light Yellow**	**Dark Yellow**	**Cream**	**White**	**CV-LY**	**CV- DY**	**CV-C**	**CV-W**	***P* value**	**LSD**
K	g kg^−1^	21.75 ± 0.40^b^	21.53 ± 0.51^b^	23.81 ±**0.81^a^**	21.49 ± 0.70^b^	43.2	42.1	45.6	41.7	0.001	0.647
P			3.12 ± 0.03^b^	3.28 ± 0.05^a^	3.24 ± 0.04^a^	3.07 ± 0.05^b^	20.5	24.4	17.9	21.6	0	0.076
Ca			0.34 ± 0.00^a^	0.35 ± 0.01^a^	0.31 ± 0.01^b^	0.33 ± 0.01^ab^	26.6	29.8	30.2	26.3	0.001	0.011
Mg		1.17 ± 0.01^a^	1.18 ± 0.02^a^	1.20 ± 0.02^a^	1.17 ± 0.02^a^	20	23.4	20.8	25	0.268	0.025
Fe	mg kg^−1^	25.91 ± 0.47^b^	27.35 ± 0.75^a^	26.45 ± 0.89^ab^	24.25 ± 0.72^c^	42.6	49	44.9	40.6	0	0.862
Zn			26.02 ± 0.35^b^	25.84 ± 0.50^b^	27.26 ±0.60^a^	24.49 ± 0.60^c^	30.8	34.6	29.7	31.2	0.002	0.806
Cu			7.97 ± 0.07^b^	8.06 ± 0.10^ab^	8.28 ±0.15^a^	7.56 ± 0.16^c^	21.7	22.6	23.7	27	0.001	0.207
Mn		6.97 ± 0.06^b^	6.93 ± 0.08^b^	7.21 ±0.11^a^	6.84 ± 0.13^b^	20.3	20.4	20.8	23.8	0.049	0.157

**Notes.**

* Means followed by different letters are significantly different by each other *P* < 0.05. LSD is at 0.05 significance level.

CV Coefficient of variation (%)

Locations: N, Niksar; K, Kazova; A, Artova, Colors: LY, Light yellow; DY, Dark yellow; C, Cream; W, White.

The mineral nutrient contents of potato accessions with four different flesh colors are given in Table 5. The mineral contents, except Mg, of potato accessions with four different flesh colors were significantly varied. The highest concentrations of minerals were obtained in cream-colored potato accessions (Table 5). The zinc levels in potatoes clones ranged from 24.49 (white colored) to 27.26 mg kg^−1^ (cream colored). The copper contents of colored potato tubers were significantly varied. Like Zn and many other mineral nutrients, the highest Cu content was recorded in cream colored (8.28 mg kg^−1^) followed by dark yellow (8.06 mg kg^−1^) and light yellow (7.97 mg kg^−1^) flesh colored potato clones. The iron content of four different colored potato clones in this study was between 27.35 (dark yellow) and 24.25 (white) mg kg^−1^ (Table 5).

The calcium content of colored potato clones ranged from 0.31 to 0.35 g kg^−1^, and the mean P content in colored potato accessions ranged between 3.07 (white colored) and 3.28 g kg^−1^ (dark-yellow colored). The magnesium content of different colored potato accessions was similar, and no significant differences were obtained. The Mg contents of potatoes varied between 1.17 (light yellow and white colored) and 1.20 g kg^−1^ (cream colored) (Table 5).

The mineral nutrient contents of potato accessions in year × location interactions are given in Table 6. The results showed that year × location interaction had a significant impact on all, except Mg and Mn, mineral contents of potato accessions. The highest contents of elements, except K and Fe, have been obtained in the second growing season at Artova. The results clearly suggested that Artova, which is located at higher altitude is the most suitable location to produce potatoes with high mineral composition.

**Table 6:** Mean nutrient contents (mean ± standard error) of potato accessions in year × location, year × color, and location × color interactions.

	K	P	Ca	Mg	Fe	Zn	Cu	Mn
	g kg^−1^				mg kg^−1^			
Year × Location								
2013xNik	28.47 ± 0.61^b^	2.90 ± 0.05^d^	0.33 ± 0.01^bc^	1.01 ± 0.01^c^	18.56 ± 0.35^e^	30.86 ± 0.56^a^	7.86 ± 0.11^b^	6.01 ± 0.08^c^
2013xKz	34.72 ± 0.50^a^	3.32 ± 0.06^b^	0.32 ± 0.01^bc^	1.20 ± 0.01^b^	16.60 ± 0.38^f^	23.51 ± 0.48^c^	7.85 ± 0.12^b^	6.55 ± 0.08^b^
2013xArt	22.66 ± 029^c^	3.28 ± 0.03^b^	0.31 ± 0.01^c^	1.35 ± 0.02^a^	20.29 ± 0.37^d^	23.88 ± 0.37^bc^	7.11 ± 0.11^c^	6.91 ± 0.09^b^
2014xNik	13.62 ± 0.31^e^	2.91 ± 0.03^d^	0.33 ± 0.01^b^	0.96 ± 0.02^d^	23.96 ± 0.60^c^	19.13 ± 0.52^d^	7.67 ± 0.17^b^	6.95 ± 0.10^b^
2014xKz	13.77 ± 0.20^e^	3.10 ± 0.04^c^	0.32 ± 0.01^bc^	1.20 ± 0.03^b^	39.84 ± 0.61^a^	25.76 ± 0.37^b^	8.65 ± 010^a^	7.72 ± 0.09^a^
2014xArt	18.57 ± 0.31^d^	3.52 ± 0.05^a^	0.39 ± 0.01^a^	1.33 ± 0.02^a^	37.60 ± 0.71^b^	32.61 ± 0.58^a^	8.76 ± 0.13^a^	7.69 ± 0.12^a^
*P* value	0.001	0.000	0.001	0.146	0.001	0.001	0.001	0.151
LSD	1.063	0.125	0.018	0.041	1.416	1.324	0.339	0.258
Year × Color								
2013xLY	28.26 ± 0.51^b^	3.10 ± 0.04^bc^	0.32 ± 0.01^c^	1.16 ± 0.01^b^	18.70 ± 0.34^d^	26.41 ± 0.48^bc^	7.62 ± 0.10^cd^	6.45 ± 0.08^c^
2013xDY	27.65 ± 0.62^b^	3.24 ± 0.06^ab^	0.33 ± 0.01^c^	1.19 ± 0.02^ab^	18.31 ± 0.41^d^	24.70 ± 0.47^cd^	7.58 ± 0.13^cd^	6.43 ± 0.09^c^
2013xC	32.05 ± 0.97^a^	3.30 ± 0.07^a^	0.30 ± 0.01^d^	1.21 ± 0.02^ab^	19.18 ± 0.60^d^	28.27 ± 0.87^a^	7.91 ± 0.20^bc^	6.63 ± 0.13^c^
2013xW	27.92 ± 0.89^b^	3.10 ± 0.07^bc^	0.32 ± 0.01^cd^	1.23 ± 0.03^a^	18.07 ± 0.59^d^	25.25 ± 0.84^bcd^	7.32 ± 0.16^d^	6.60 ± 0.14^c^
2014xLY	15.20 ± 0.27^c^	3.13 ± 0.03^bc^	0.35 ± 0.01^ab^	1.18 ± 0.02^b^	33.12 ± 0.63^b^	25.65 ± 0.49^bc^	8.32 ± 0.11^ab^	7.48 ± 0.08^a^
2014xDY	15.48 ± 0.42^c^	3.32 ± 0.06^a^	0.36 ± 0.01^a^	1.18 ± 0.03^ab^	36.37 ± 1.02^a^	26.96 ± 0.87^ab^	8.53 ± 0.15^a^	7.43 ± 0.12^a^
2014xC	15.57 ± 0.40^c^	3.18 ± 0.05^abc^	0.33 ± 0.01^bc^	1.18 ± 0.03^ab^	33.73 ± 1.27^b^	26.25 ± 0.82^bc^	8.65 ± 0.21^a^	7.79 ± 0.16^a^
2014xW	15.05 ± 0.42^c^	3.04 ± 0.08^c^	0.34 ± 0.01^abc^	1.11 ± 0.03^c^	30.43 ± 1.05^c^	23.73 ± 0.86^d^	7.81 ± 0.28^cd^	7.07 ± 0.21^b^
*P* value	0.000	0.342	0.900	0.006	0.000	0.001	0.563	0.075
LSD	0.914	0.107	0.015	0.035	1.218	1.138	0.292	0.222
Location × Color								
NikxLY	20.94 ± 0.93^cd^	2.85 ± 0.04^f^	0.33 ± 0.01^b–e^	0.99 ± 0.01^e^	22.26 ± 0.60^e^	25.81 ± 0.74^bc^	7.79 ± 0.14^bcd^	6.51 ± 0.10^d^
NilxDY	19.09 ± 0.82^e^	3.01 ± 0.07^ef^	0.35 ± 0.01^abc^	0.96 ± 0.02^e^	21.82 ± 0.71^ef^	22.73 ± 0.76^de^	7.84 ± 0.62^bcd^	6.46 ± 0.14^d^
NikxC	24.42 ± 1.63^b^	2.95 ± 0.08^f^	0.30 ± 0.01^e^	1.02 ± 0.03^e^	20.02 ± 0.80^fg^	27.32 ± 1.40^ab^	7.73 ± 0.27^bcd^	6.53 ± 0.19^d^
NikxW	21.35 ± 1.39^cd^	2.87 ± 0.09^f^	0.32 ± 0.01^cde^	0.98 ± 0.03^e^	19.28 ± 0.79^g^	24.08 ± 1.25^cde^	7.58 ± 0.29^cd^	6.40 ± 0.15^d^
KzxLY	24.15 ± 0.87^b^	3.19 ± 0.05^cd^	0.33 ± 0.01^b–e^	1.19 ± 0.01^d^	27.17 ± 0.97^cd^	24.70 ± 0.45^cd^	8.21 ± 0.12^bc^	7.12 ± 0.11^bc^
KzxDY	24.01 ± 1.16^b^	3.19 ± 0.08^cde^	0.32 ± 0.01^cde^	1.18 ± 0.02^d^	29.28 ± 1.45^ab^	25.37 ± 0.62^bc^	8.10 ± 0.15^bc^	6.98 ± 0.12^c^
KzxC	26.46 ± 1.64^a^	3.47 ± 0.07^ab^	0.31 ± 0.01^de^	1.25 ± 0.02^cd^	28.97 ± 1.87^abc^	25.29 ± 0.87^bc^	9.16 ± 0.21^a^	7.50 ± 0.16^ab^
KzxW	22.62 ± 1.42^bc^	3.04 ± 0.08^def^	0.31 ± 0.01^de^	1.21 ± 0.03^d^	27.90 ± 1.56^bcd^	22.19 ± 0.74^e^	7.68 ± 0.22^bcd^	7.08 ± 0.22^bc^
ArtxLY	20.17 ± 0.36^de^	3.32 ± 0.04^bc^	0.34 ± 0.01^a–d^	1.31 ± 0.02^bc^	28.30 ± 0.79^bc^	27.53 ± 0.54^ab^	7.91 ± 0.12^bcd^	7.26 ± 0.10^abc^
ArtxDY	21.45 ± 0.42^cd^	3.64 ± 0.07^a^	0.37 ± 0.01^a^	1.40 ± 0.02^a^	30.93 ± 1.40^a^	29.45 ± 1.03^a^	8.24 ± 0.18^b^	7.35 ± 0.14^abc^
ArtxC	20.56 ± 0.55^cde^	3.23 ± 0.05^bc^	0.33 ± 0.01^cde^	1.33 ± 0.03^b^	30.36 ± 1.40^ab^	29.17 ± 0.68^a^	7.96 ± 0.24^bcd^	7.60 ± 0.20^a^
ArtxW	20.49 ± 0.73^cde^	3.31 ± 0.09^bc^	0.36 ± 0.01^ab^	1.32 ± 0.04^bc^	25.57 ± 1.29^d^	27.19 ± 0.98^ab^	7.42 ± 0.32^d^	7.03 ± 0.27^c^
*P* value	0.001	0.002	0.109	0.010	0.004	0.000	0.012	0.609
LSD	1.117	0.131	0.018	0.043	1.488	1.391	0.357	0.271

**Notes.**

Means followed by different letters are significantly different by each other *P* < 0.05. LSD is at 0.05 significance level.

Locations: N: Niksar, K:Kazova, A:Artova, Colors: LY: Light yellow; DY: Dark yellow; C: Cream; W: White

The mineral nutrient contents of potato accessions in year × color interactions are given in Table 6. The changes in K, Mg, Fe and Zn in the interaction of year × color were statistically significant (*p* < 0.01), while the interaction had no significant impact on P, Ca, Cu and Fe contents of potato accessions. The highest K (32.05 g kg^−1^) and Zn contents (28.27 mg kg^−1^) were obtained in the first growing season for cream colored potato accessions. The white colored potato accession were rich in Mg content in the first growing season.

The mineral nutrient contents of potato accessions in location × color interactions are given in Table 6. The results showed that the mineral contents (except Ca and Mn) significantly varied with the location × color interaction. The highest mean K content (26.46 g kg^−1^) was obtained in Kazova × cream interaction, while the lowest mean K content (19.09 g kg^−1^) was recorded in Niksar × dark yellow interaction. The contents of P, Ca, Mg, Fe and Zn were at the highest in Artova × dark yellow interactions (Table 6).

The mineral nutrient contents of potato accessions in year × location × color interactions are given in Table 6. The highest mineral contents (except K and Mn) of potato accessions in year × location × color interactions were determined in the second growing season × Artova × dark yellow interactions. The highest mean K content (38.06 g kg^−1^) was obtained in the cream potato cultivars grown in Kazova during the first growing season (Table 7). Although average K content of cream colored potato accessions was higher compared to the other three colored potatoes, there was only one cream colored potato line in the first five potato accessions with the highest K content, which were A1/9 (light yellow), A2/127 (light yellow), A10/15 (cream), A3/108 (dark yellow), and A3/26 (light yellow). The Mg content of dark yellow (in 2014) and white color potato (in 2013) accession grown in Artova was significantly higher compared to the other potatoes grown in both growing seasons (Table 7). The first five potato accessions with the highest Mg content were A2/11 (dark yellow), A1/58 (white), A9/8 (dark yellow), Granola (dark yellow) and A5/98 (cream) (Data S1).The potato accessions with the highest Fe contents were obtained Artova × dark yellow × 2014 (42.63 mg kg^−1^), Kazova × dark yellow × 2014 (41.96 mg kg^−1^) and Kazova × cream × 2014 (41.60 mg kg^−1^) interactions (Table 7). Although mean Fe content was around 40 mg kg^−1^, the mean Fe content of some accessions such as A9/4 (61.66 mg kg^−1^), A3/108 (60.60 mg kg^−1^) and A3/26 (57.34 mg kg^−1^) was almost 50% higher than the average Fe content Data S1.

**Table 7:** Mean nutrient contents (mean ± standard error) of potato accessions in year × location × color interactions.

Year × Loc Color	K	P	Ca	Mg	Fe	Zn	Cu	Mn
	g kg^−1^				mg kg^−1^			
1xNxLY	27.71 ± 0.88^d^	2.80 ± 0.07^g^	0.33 ± 0.01^d–g^	0.98 ± 0.02^fg^	19.30 ± 0.56^f–i^	31.87 ± 0.85^bc^	7.79 ± 0.15^d–h^	5.82 ± 0.11^k^
1xNxDY	25.99 ± 0.91^de^	3.07 ± 0.12^d–g^	0.36 ± 0.02^b–e^	1.01 ± 0.02^fg^	19.11 ± 0.71^ghi^	27.70 ± 0.85^de^	8.03 ± 0.21^def^	6.16 ± 0.18^ijk^
1xNxC	34.99 ± 1.64^b^	2.95 ± 0.15^efg^	0.30 ± 0.02^fg^	1.07 ± 0.04f	18.39 ± 0.75^ghi^	34.08 ± 1.54^ab^	7.75 ± 0.31^d–h^	6.06 ± 0.20^jk^
1xNxW	28.56 ± 1.90^d^	2.90 ± 0.16^fg^	0.31 ± 0.01^efg^	1.05 ± 0.05^f^	17.39 ± 0.66^hi^	29.94 ± 1.54^cd^	7.92 ± 0.29^d–g^	6.33 ± 0.20^h–k^
1xKxLY	34.54 ± 0.75^b^	3.30 ± 0.10^cd^	0.34 ± 0.01^c–f^	1.17 ± 0.02^e^	16.21 ± 0.56^i^	23.79 ± 0.75^f–i^	7.80 ± 0.18^d–h^	6.49 ± 0.13^g–j^
1xKxDY	34.77 ± 0.93^b^	3.31 ± 0.14^cd^	0.32 ± 0.01^d–g^	1.18 ± 0.02^e^	16.60 ± 0.77^i^	23.79 ± 0.86^f–i^	7.71 ± 024^d–h^	6.46 ± 0.16^g–k^
1xKxC	38.06 ± 1.13^a^	3.67 ± 0.08^ab^	0.30 ± 0.02^fg^	1.28 ± 0.02^b–e^	16.35 ± 0.85^i^	24.05 ± 1.43^fgh^	9.02 ± 0.33^abc^	6.95 ± 0.17^e–h^
1xKxW	31.55 ± 1.30^c^	3.04 ± 0.09^d–g^	0.32 ± 0.02^efg^	1.22 ± 0.05^cde^	18.10 ± 1.22^ghi^	21.38 ± 1.10^h–k^	7.00 ± 0.19^gh^	6.49 ± 0.21^g–j^
1xAxLY	22.54 ± 0.49^fg^	3.22 ± 0.04^c–f^	0.30 ± 0.01^fg^	1.34 ± 0.02^ab^	20.57 ± 0.54^fgh^	23.48 ± 0.56^f–i^	7.25 ± 0.16^fgh^	7.05 ± 0.13^d–g^
1xAxDY	22.13 ± 0.46^fg^	3.34 ± 0.05^cd^	0.31 ± 0.01^efg^	1.37 ± 0.03^ab^	19.24 ± 0.58^f–i^	22.72 ± 0.57^g–j^	7.02 ± 0.17^gh^	6.67 ± 0.13^f–j^
1xAxC	23.11 ± 0.68^fg^	3.28 ± 0.08^cde^	0.29 ± 0.01^g^	1.30 ± 0.05^bc^	22.80 ± 1.12^def^	26.69 ± 0.87^def^	6.96 ± 0.28^h^	6.89 ± 0.27^e–h^
1xAxW	23.65 ± 0.85^ef^	3.36 ± 0.11^cd^	0.32 ± 0.01^d–g^	1.42 ± 0.05^a^	18.72 ± 1.12^ghi^	24.42 ± 0.87^e–h^	7.03 ± 0.32^gh^	6.98 ± 0.28^e–h^
2xNxLY	14.03 ± 0.58^j^	2.90 ± f.05^g^	0.34 ± 0.01^c–f^	1.01 ± 0.02^fg^	25.22 ± 0.97^d^	19.75 ± 0.83^jkl^	7.79 ± 0.23^d–h^	7.21 ± 0.15^c–f^
2xNxDY	12.44 ± 0.38^j^	2.96 ± 0.07^efg^	0.34 ± 0.02^c–f^	0.92 ± 0.04^g^	24.54 ± 1.11^de^	17.76 ± 0.82l	7.65 ± 0.34^e–h^	6.75 ± 0.19^f–i^
2xNxC	13.86 ± 0.67^j^	2.95 ± 0.08^efg^	0.30 ± 0.01^fg^	0.97 ± 0.04^fg^	21.66 ± 1.35^efg^	20.56 ± 1.56^i–l^	7.70 ± 0.44^d–h^	7.00 ± 0.31^d–h^
2xNxW	14.14 ± 0.51^j^	2.83 ± 0.05^g^	0.32 ± 0.01^d–g^	0.92 ± 0.04^g^	21.18 ± 1.35^efg^	18.23 ± 1.15^kl^	7.24 ± 0.49^f–h^	6.47 ± 0.23^g–k^
2xKxLY	13.76 ± 0.25^j^	3.08 ± 0.04^d–g^	0.33 ± 0.01^d–g^	1.21 ± 0.02^cde^	38.12 ± 0.87^b^	25.61 ± 0.46^efg^	8.61 ± 0.16^a–d^	7.76 ± 0.14^abc^
2xKxDY	13.25 ± 0.42^j^	3.06 ± 0.07^d–g^	0.32 ± 0.01^d–g^	1.19 ± 0.02^de^	41.96 ± 1.37^a^	26.95 ± 0.85^def^	8.50 ± 0.15^b–e^	7.50 ± 0.14^b–e^
2xKxC	14.86 ± 0.59^ij^	3.27 ± 0.10^cde^	0.32 ± 0.02^d–g^	1.22 ± 0.04^cde^	41.60 ± 1.58^a^	26.53 ± 0.98^ef^	9.29 ± 0.26^ab^	8.05 ± 0.24^ab^
2xKxW	13.68 ± 0.59^j^	3.03 ± 0.15^d–g^	0.30 ± 0.02^fg^	1.19 ± 0.05^de^	37.70 ± 1.02^b^	22.30 ± 0.98^g–j^	8.36 ± 0.35^cde^	7.67 ± 0.36^a–d^
2xAxLY	17.81 ± 0.40^h^	3.41 ± 0.06^bc^	0.38 ± 0.01^bc^	1.29 ± 0.02^bcd^	36.03 ± 0.94^b^	31.59 ± 0.71^bc^	8.56 ± 0.16^b–e^	7.46 ± 0.14^b–e^
2xAxDY	20.76 ± 0.70^g^	3.94 ± 0.13^a^	0.42 ± 0.01^a^	1.44 ± 0.04^a^	42.63 ± 1.58^a^	36.18 ± 1.50^a^	9.45 ± 0.20^a^	8.04 ± 0.22^ab^
2xAxC	18.01 ± 0.57^h^	3.31 ± 0.08^cd^	0.36 ± 0.02^bcd^	1.36 ± 0.04^ab^	37.93 ± 1.65^b^	31.66 ± 0.85^bc^	8.97 ± 0.30^abc^	8.31 ± 0.21^a^
2xAxW	17.33 ± 0.83^hi^	3.27 ± 0.15^cde^	0.40 ± 0.02^ab^	1.21 ± 0.06^cde^	32.43 ± 1.37^c^	29.97 ± 1.35^cd^	7.82 ± 0.56^d–h^	7.08 ± 0.46^d–g^
*P* value	0.001	0.017	0.350	0.004	0.013	0.173	0.031	0.001
LSD(0.05)	1.572	0.185	0.026	0.06	2.093	1.957	0.502	0.381

**Notes.**

Means followed by different letters are significantly different by each other *P* < 0.05.

Year: 1, 2013 and 2, 2014.

Locations: N, Niksar; K, Kazova; A, Artova, Colors: LY, Light yellow; DY, Dark yellow; C, Cream; W, White.

## Discussion

The findings obtained in three different geographical locations clearly revealed the significant diversity for mineral nutrient contents of these potato accessions. Potassium is one of the most essential nutrients, without which plants cannot grow in soils with insufficient K supply (Marschner, 2012). The potassium concentration of potatoes is in general quite high compared to many other crops, ranging from 150 to 1386 mg 100 g^−1^ FW (Nassar et al., 2012). In contrast to most of the mineral nutrients, the K content of potatoes grown in Artova was lower than the other two locations, and low K content of potato accessions in Artova can be attributed to the low K content of soils in the experimental field (Table 1). High K content of potato clones in the study is in accordance with the report of Navarre, Goyer & Shakya (2009) who indicated that potatoes may provide up to 18% of the recommended daily allowance of K, 6% of Fe, P and Mg, and 2% of Ca and Zn. The content of mineral nutrients in four different flesh colored potato accessions are in accordance with the findings of Singh et al. (2022a) who determined the macro and micronutrient contents of one hundred tetraploid potato accessions obtained from the Indian Council of Agricultural Research, Central Potato Research Institute, Shimla, India. The concentrations of micronutrients in Indian potato varieties were as follows; Fe was between 23.60–76.90 mg kg^−1^, Zn between 6.80–36.00 mg kg^−1^, Cu between 3.80–23.10 mg kg^−1^ and Mn between 7.20–28.40 mg kg^−1^. The concentration of Ca ranged from 0.2 to 1.0 g kg^−1^, Mg from 0.4 to 2.9 g kg^−1^, P from 1.3 to 3.9 g kg^−1^ and K from 4.8–26.1 g kg ^−1^. The differences in mineral nutrient contents among various clones of potato have been reported by Andre et al. (2007), Subramanian et al. (2017) and some other researchers. The differences in mineral nutrient compositions between potato accessions in three different locations (Table 5) could be attributed to the adaptation mechanisms of potato accession to the growing conditions (Leonel et al., 2017). The adaption mechanisms described by Leonel et al. (2017) are changes in morphology, architecture of the root system and physiological characteristics of roots. Navarre, Goyer & Shakya (2009) stated that location, stage of development, soil properties (pH, organic matter content, *etc*.,) timing and amount of fertilizers, irrigation, and weather are the most influential factors on the mineral composition of potatoes. The differences in soil properties, altitude, and climate between the experimental site (Table 1 and 2) may cause the differences in mineral nutrient content of potato accessions grown during these experiments. The findings of Burgos et al. (2007) support the significant differences in the same mineral nutrients of potato tubers grown in different locations. Burgos et al. (2007) reported that different mineral contents for the same genotypes grown in different locations were due to changes in environmental conditions.

A high range of mineral nutrient contents among the potato accessions indicates a breeding potential for high tuber mineral contents (Subramanian et al., 2017). These findings revealed that cream-colored potatoes contain more mineral nutrients than light yellow, dark yellow and white color potatoes. Cream-colored potatoes had the highest K contents, while the K contents of white-colored potatoes were at the lowest level. Potato is usually known for its high K content and Lisinska & Leszczynski (1989) stated that the K content of potatoes is higher than the K content of bananas, which is always suggested by dieticians. Similar to the K content, the highest P, Mg, Zn, Cu and Mn contents were recorded in cream-colored potatoes (Table 5). The mean P content in colored potato accessions (between 3.07 and 3.24 g kg^−1^) was sufficient (Table 5) for the daily P requirement (800–1,000 mg) for a human being (Navarre, Goyer & Shakya, 2009). The P content in different locations was significantly higher in Artova, where many other nutrients were at their highest levels. The P content of potatoes grown in three different locations of Sao Paola, Brazil (ranging between 0.55 and 1.05 g kg^−1^) was lower (Souza et al., 2022) than the P contents recorded in this study. The researchers indicated that high nitrogen applications reduced the P content of tubers while increasing tuber size.

Magnesium is important to activate more than 300 enzymes in the human body (Al Alawi, Majoni & Falhammar, 2018), therefore, cultivation of Mg rich potato cultivars is needed to supply the Mg demands of human body. The findings highlighted the significance of cream-colored potato cultivars in breeding efforts to increase Mg concentration in potato tubers. In contrast to other nutrients, the Ca content of cream-colored potatoes was lower compared to the other three colored potatoes. Brown et al. (2012) reported that the Ca content of potatoes increases the resistance of potato tubers against the internal brown spot, heat necrosis, infection by the soft rot pathogen *Pectobacterium* spp., heat tolerance, and freezing tolerance; therefore, low Ca contents of cream-colored potatoes can be considered low resistance to aforementioned stress factors. Subramanian et al. (2017) showed that the Ca content of potato tubers was between 0.1 and 0.7 mg g^−1^. The Ca content of potato clones tested in this study was within the range stated by Subramanian et al. (2017).

Significant differences were found between the Zn content of colored potatoes (Table 5). Zinc is an essential micronutrient needed in a human diet, because the Zn serves as the cofactor for the various enzymes in the cell. Therefore, children and pregnant women who experience a lack of adequate Zn, especially in developing countries, face a severe health problem related to Zn deficiency (Brown et al., 2011). The Zn content of wheat in the world is 31.84 mg kg^−1^ (Wang et al., 2020), which is slightly high compared to the average Zn contents of the potato accessions used in this study. Although the mean Zn content of potato accessions ranges from 24.49 (white) and 27.26 (cream) (Table 5), some potato lines, such as A2/11 (53.94 mg kg^−1^ in Artova, 2014), A9/4 (49.15 mg kg^−1^ in Artova, 2014) and A3/234 (48.03 mg kg^−1^ in Artova, 2014) contain much higher Zn contents than the average values Data S1. The potato accessions with the highest Zn content are ideal parental material in biofortification to reduce the Zn deficiency problem (Singh et al., 2022b).

Similar to the findings of this study, Dugo et al. (2004) showed that the Cu content of yellow-fleshed potatoes was high. The Cu contents of potato clones used in this study area are within the range of Cu contents in potato clones (0.23–11.9 mg/kg FW) reported by Randhawa et al. (1984) and Casañas-Rivero et al. (2003). Brown et al. (2010) identified potatoes as a very important source of Fe for the diet. The researchers reported that the range of mean Fe content for 33 clones grown in 12 different environments was between 17 and 62 ug per gram of dry weight.

The range of Fe content in this study is close to the lower Fe contents reported in Brown et al. (2010). The baseline for Fe in potatoes was reported between 0.25 and 0.83 mg/100 g FW (Burgos et al., 2007), while the mean Fe contents of potato accessions are higher than the baseline indicated by Burgos et al. (2007). The Fe contents of potatoes in various studies may differ due to the differences in sample preparation for the Fe analysis (Brown et al., 2010). In the current study, the soil was completely removed, tubers were peeled, and surface cleaning was carried out with HCl prior to the analysis. For example, Casañas-Rivero et al. (2003) did not use acid to clean the surface and reported the mean Fe content of “native” Canary Islands and imported varieties as 94 and 112 µg/g DW, respectively. The Fe contents reported by Casañas-Rivero et al. (2003) were somewhat higher than the Fe contents of potato accessions grown in three locations. The significant differences in Fe content, as in other micronutrients, can be attributed to the differences in soil properties as well as micronutrient contents of the experimental sites. The findings of Souza et al. (2022) on differences in Fe, Zn, Cu, and Mn contents of potato grown in three different locations are in agreement with the current study.

Phosphorus is one of the most important plant nutrients for many crops, as it is the main component of several co-enzymes and phospholipids (Raghothama, 1999); it is also one of the main mineral nutrient of the potato tubers (Navarre, Goyer & Shakya, 2009). Sanchez-Castillo et al. (1998) stated that the P content in potatoes ranges from 1300 to 6000 g g^−1^ DW. The Commonwealth Potato Collection *Solanum* species had a phosphorus content ranging from 2.4 to 5.2 mg g^−1^ (Subramanian et al., 2017). The P content of all potato accessions was within the range of P content as determined by the aforementioned studies. The magnesium content of potatoes reported by Lal et al. (2020) was between 16 to 40 mg/100 g FW. The magnesium content of selected Commonwealth Potato Collection accessions, representing the eco-geographical distribution of wild potatoes, was between 0.8 and 2.2 mg g^−1^ (Subramanian et al., 2017). The Mg content of 67 potato clones used in this study is within the ranges reported in previous studies.

## Conclusions

The content of eight mineral nutrients (K, P, Ca, Mg, Fe, Zn, Cu and Mn) of fifty-eight tetraploid potato accessions with four different flesh colors and grown in three different locations were evaluated. Changes in environmental conditions (soil and climate) significantly affected the mineral nutrient composition of the same clones. The mineral nutritional compositions of the potato cultivars grown in this study differed significantly. Overall, cream and dark yellow flesh colored potato clones contain significantly higher mineral nutrients. The mineral nutrients (except K and Cu) in potato clones were higher in Artova due to lower soil pH, calcium carbonate and clay content compared to the other two locations. The results revealed that Kazova is suitable to cultivate potatoes with high potassium content. Finally, these results suggest that potatoes with high mineral nutrients can be produced in high altitudes when using cream and dark yellow flesh colored potato clones rather than lowland locations using light yellow and white colored potatoes. In addition, the potato accessions with the highest nutrient contents might be useful in breeding to develop biofortified potato varieties.

##  Supplemental Information

10.7717/peerj.15262/supp-1 Data S1 Raw data K (mg/kg): Potassium; P: Phosphorus; Ca: Calcium; Mg; Magnesium; Fe: Iron; Zn: Zinc; Cu: Cupper; Mn: Manganese; Color Code: 1: light yelley; Color Code 2: Dark yellow; Color Code 3: Cream; Color Code 4: White Location Code 1:Niksar; Location Code 2:Kazova; Location Code 3: Artova

10.7717/peerj.15262/supp-2 Data S2 The results for chemical analysis of potato tubers All data obtained in statistical analysis; correlation test and ANOVA analysis.

## Additional Information and Declarations

### Data Availability

The following information was supplied regarding data availability: The raw measurements are available in the Supplemental Files.
